# Eosinophilic Myocarditis: A Challenging Diagnosis

**DOI:** 10.7759/cureus.24371

**Published:** 2022-04-22

**Authors:** Zahra Nassereddine, Fida Charif, Claudette Najjar, Ziad Neghawi, Mohamad Saab

**Affiliations:** 1 Cardiology Division, Beirut Cardiac Institute, Beirut, LBN; 2 Pulmonary Critical Care, Cardiovascular Division, Beirut Cardiac Institute, Beirut, LBN; 3 Radiology Division, Beirut Cardiac Institute, Beirut, LBN; 4 Cardiovascular Surgery Division, Beirut Cardiac Institute, Beirut, LBN

**Keywords:** hypereosinophilic syndromes, endomyocardial biopsy, cardiac magnetic resonance imaging, peripheral eosinophilia, eosinophilic myocarditis

## Abstract

Eosinophilic myocarditis (EM) is a rare subtype of myocarditis that is characterized by eosinophilic infiltration of the myocardium and is associated with peripheral eosinophilia in most cases. The diagnosis is suspected in the presence of acute myocarditis and peripheral eosinophilia and is usually confirmed by endomyocardial biopsy (EMB) before starting steroid therapy. Here, we present a case of severe idiopathic eosinophilic myocarditis in a young man with a history of asthma and peripheral eosinophilia. He was treated with high-dose steroids despite negative EMB, and we noted a dramatic improvement in cardiac function. Our case highlights the importance of cardiac magnetic resonance (CMR) and clinical judgment in establishing the diagnosis of EM irrespective of the histopathologic result.

## Introduction

Eosinophilic myocarditis (EM) is a spectrum of myocardial diseases that can be acute or chronic. In its acute form, the patient may present with rapidly progressive necrotizing EM that is associated with a high mortality rate while the chronic form appears as restrictive cardiomyopathy with hypereosinophilic syndromes (HES) resulting in heart failure within a few years [1-2]. EM is most commonly reported in patients with hypersensitivity reactions; however, it has been also described in antineutrophil cytoplasmic antibody (ANCA)-associated vasculitis, hypereosinophilic syndromes, sarcoidosis, parasitic infections, and certain neoplasms [3]. EM is a term used to describe eosinophilic myocarditis with an unknown cause. Appropriate workup is essential to rule out the most common treatable causes. Here, we present a case of EM with a challenging diagnosis; we discuss its main clinical presentations and suggest a different diagnostic approach.

## Case presentation

**Figure 1:**
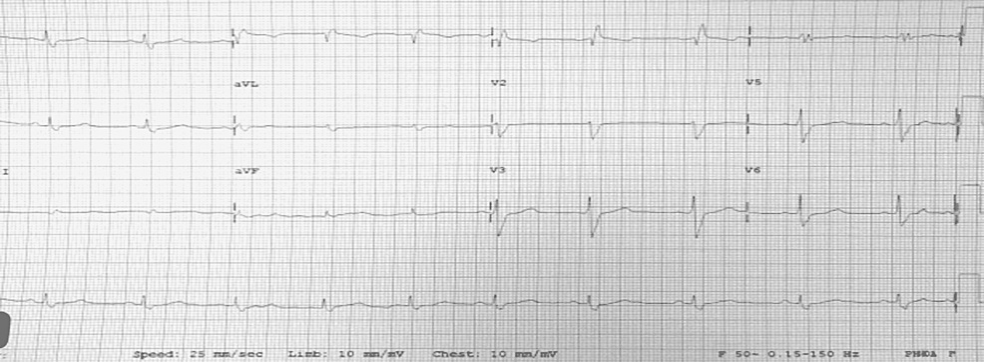
Electrocardiogram shows a right bundle branch block and a left anterior fascicular block

We present the case of a 32-year-old man who was admitted to another institution for shortness of breath, cough, and peripheral edema two weeks following an upper respiratory tract infection. He was found to have severe left ventricular failure for which he was transferred to our institution for further workup and management. Our patient is known to have mild intermittent asthma and receives daily low-dose inhaled corticosteroids; he had also a history of frequent traveling to Africa during the last two years. Upon presentation, the patient was critically ill, diaphoretic, and dyspneic. His vital signs were as follows: oxygen saturation at room air was 82%, systolic blood pressure 90 mmHg, and heart rate 100 beats per min. His physical examination was remarkable for bilateral crackles on lung auscultation and peripheral lower limbs edema. An electrocardiogram showed sinus rhythm with a left posterior fascicular block and an incomplete right bundle branch block (Figure 1). Blood workup showed severe peripheral eosinophilia; absolute eosinophil count was 7900 cells/microL. Troponin and brain natriuretic peptides (BNP) levels were elevated (Table 1).

**Table 1:** The initial blood tests and the reference ranges for each test BNP: brain natriuretic peptides

Blood tests	value	Reference range
White blood cells (/microL)	17400	4000-11000
Eosinophils percentage (%)	45.8	0-7
Absolute eosinophils count (/microL)	7970	0-700
Hemoglobin (g/dL)	15.1	13.5-18
Creatinine (mg/dL)	1.16	0.6-1.3
BNP (pg/mL)	546	<100
Troponin T (ng/mL)	0.597	0-0.014

**Figure 2:**
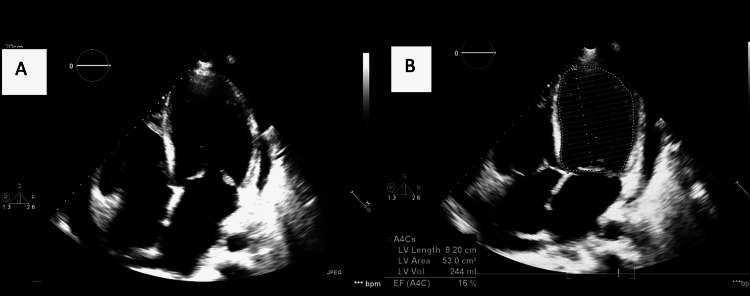
A. Transthoracic echocardiography (apical four-chamber view) shows severe LV dilation and dysfunction; B. Estimated EF using Simpson’s method LV: left ventricle, EF: ejection fraction

**Figure 3:**
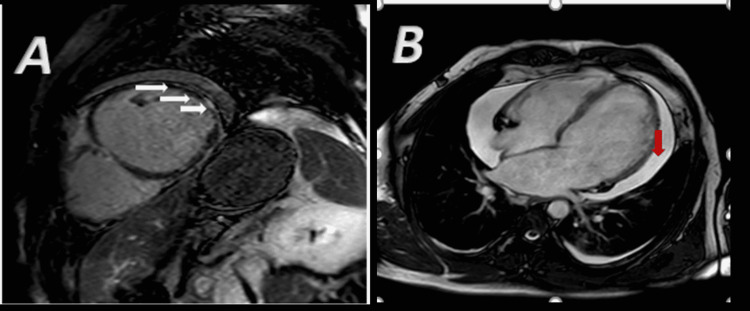
CMR demonstrates diffuse subepicardial gadolinium enhancement (white arrows) in short axis (A) and apical four-chamber view (B) compatible with myocarditis Note the presence of pericardial effusion (red arrow). CMR: cardiac magnetic resonance

Transthoracic echocardiography (TTE) showed a severely dilated left ventricle with severe systolic dysfunction and an estimated ejection fraction (EF) of around 15% (Figure 2; Video 1) as compared to the mild left ventricle (LV) dilation and dysfunction on TTE done two weeks earlier. There was also mild pericardial effusion and severe impairment of right ventricle (RV) systolic function. Coronary angiography was completely normal. The subsequent CMR showed diffuse subepicardial and mid-wall late gadolinium enhancement (LGE) mainly at the level of the septum and lateral wall, suggesting diffuse edema compatible with severe acute myocarditis (Figure 3). The RV again appeared dilated with severe systolic dysfunction. The patient denied a history of new drug or herbal product intake. A complete infectious workup, including the most common endemic parasites in Africa, namely, schistosomiasis, strongyloidiasis, Toxocara canis, and echinococcus, was negative. A whole-body computed tomography was also performed and was irrelevant. We noted the absence of sinus or skin lesions suggestive of vasculitis. C-ANCA and P-ANCA were negative. A bone marrow aspirate showed hypercellular bone marrow rich with mature eosinophils. Molecular testing for FIP1L1-PDGFR was negative.

**Video 1:** Initial transthoracic echocardiography (apical four-chamber view)

**Figure 4:**
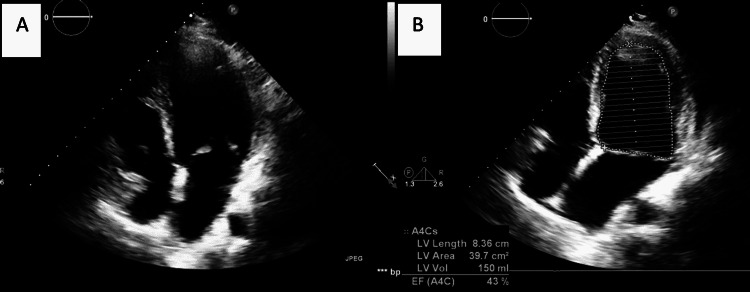
A. Transthoracic echocardiography (apical four-chamber view) shows an improvement in LV function; B. Estimated EF using Simpson’s method LV: left ventricle, EF: ejection fraction

On the second day of admission, he developed an electrocardiographic instability with episodes of sinus bradycardia and intermittent rapid atrial fibrillation associated with hypotension. Hence, an endomyocardial biopsy was done and showed myocardial necrosis with lymphocyte predominance; there were no eosinophilic infiltrates and no signs of vasculitis. Even in the absence of histopathologic confirmation, the diagnosis of idiopathic eosinophilic myocarditis was considered after having previously excluded the most common helminthic infections associated with peripheral eosinophilia, especially strongyloidiasis. He received a course of pulse steroids therapy (1 g of methylprednisolone daily for three days). Seven days later, we noted a dramatic improvement in left ventricular ejection fraction (LVEF) (EF estimated at 45%) and normalization of RV systolic function on TTE (Figure 4; Video 2). We also noted a progressive shrinking of LV dimensions and LV wall thickness along with the resolution of pericardial effusion. Our patient was discharged on day 19 on a reduced oral corticosteroids dose that was tapered over a period of three months. We noted a normal EF at the nine-month- follow-up, and he is still followed very closely.

**Video 2:** Transthoracic echocardiography, apical four-chamber view

## Discussion

EM is a rare subtype of myocarditis secondary to eosinophilic infiltration of the myocardium. The severity of the cardiac disease depends on the degree of myocardial involvement. It ranges from a mild asymptomatic form to severe fulminant myocarditis or acute necrotizing eosinophilic myocarditis [1]. It can also present as a chronic process resulting in restrictive cardiomyopathy [2]. EM is the cardiac manifestation of different conditions with or without peripheral eosinophilia. The most common one is a drug-induced hypersensitivity reaction [3]; it is called hypersensitivity myocarditis in this case. The causative drugs are mainly antibiotics (e.g. minocycline, beta-lactam), but reactions with other drugs were also reported [3]. ANCA-associated vasculitis, hypereosinophilic syndromes, parasitic infections, and certain types of solid or hematologic malignancies have also been reported as frequent causes of EM.

Although EMB is required for the definitive diagnosis, some cases of non-histologically proven myocarditis were also reported [3]. The sensitivity of endomyocardial biopsy based on autopsy specimens was estimated to be around 54% in case of patchy myocardial infiltration [4]. Some authors suggest using an MRI-guided endocardial biopsy to detect the areas of infiltration and increase the likelihood of the diagnosis [5].

Early in our diagnostic approach, eosinophilic granulomatosis with polyangeitis (EGPA) was considered a possible cause of EM in our patient. However, EGPA, according to the American College of Rheumatology, requires the presence of four out of six criteria: asthma, migratory infiltrates in the lung, paranasal sinus abnormalities, mono or polyneuropathy, peripheral blood eosinophilia (greater than 10% total leucocyte count), and eosinophilic tissue infiltrates in the biopsy [6]. Our patient had only three clinical criteria with the absence of other organ involvement making EGPA less likely.

HES is one of the causes of peripheral hypereosinophilia. HES is defined by persistent primary eosinophilia of more than 1500 eosinophils/mm3 with end-organ damage due to eosinophils infiltration in the absence of an evident cause of hypereosinophilia. Cardiac complications occur in 40%-50% of cases and include three stages: acute necrotic stage (4-5 weeks), thrombosis (10 months), and ultimately fibrosis in two to three years [7]. Restrictive cardiomyopathy with endomyocardial thickening, the formation of apical thrombus, and atrioventricular valves regurgitation are typical findings. There are many subtypes of HES; cardiac involvement is observed mainly in the myeloproliferative subtype, which is characterized by the presence of specific clonal chromosomal aberrations, mainly FIP1L1-PDGFRA fusion, and has a completely different treatment based on the tyrosine kinase activity inhibitor [8]. The lymphocytic variant, characterized by the presence of a phenotypically aberrant lymphocyte clone, is responsive to steroids. Similar syndromes, probably secondary to a helminthic infection, Loeffler endocarditis, and endomyocardial fibrosis, are typically seen in temperate climates and tropical areas, respectively.

Our patient could have the initial stage of HES with negative molecular studies for myeloproliferative and lymphocytic variants, however, the diagnosis of HES remains less likely. Therefore, idiopathic EM was considered the most likely diagnosis based on the clinical and paraclinical presentation of acute myocarditis along with the severe peripheral eosinophilia, CMR findings, and absence of an alternative diagnosis. The negative EMB in our patient is due to the non-homogenous infiltration of the myocardium.

The treatment of EM depends on the underlying disease [2-3]. The cessation of the offending drug is indicated in cases of drug-induced hypersensitivity in addition to steroid therapy in severe cases. In patients with EGPA or other types of vasculitis, the treatment is based on corticosteroids combined with other immunosuppressive therapy in more advanced cases (e.g. cyclophosphamide, azathioprine) while anti-parasitic drugs should be added to corticosteroids when EM is related to parasitic infections. EM in the setting of HES is treated according to its subtypes with imatinib and steroids initially in myeloproliferative disorders associated with fusion genes FIP1L1, PDGFRA, and PDGFRB and with steroids alone in the lymphocytic subtype [9]. Furthermore, corticosteroids were also used in the treatment of idiopathic EM with no clear recommendations regarding the treatment regimen.

## Conclusions

We described a case of eosinophilic myocarditis with a negative endomyocardial biopsy. The diagnosis of EM was confirmed retrospectively after LV improvement on steroids therapy and after excluding the most common etiologies of peripheral hypereosinophilia. Early diagnosis and treatment are crucial in improving patient outcomes. Although EMB is essential for definitive diagnosis, it is not highly sensitive and can delay treatment initiation. Thus, a new diagnostic approach based on the criteria of CMR, peripheral eosinophilia, and clinical judgment is needed.
